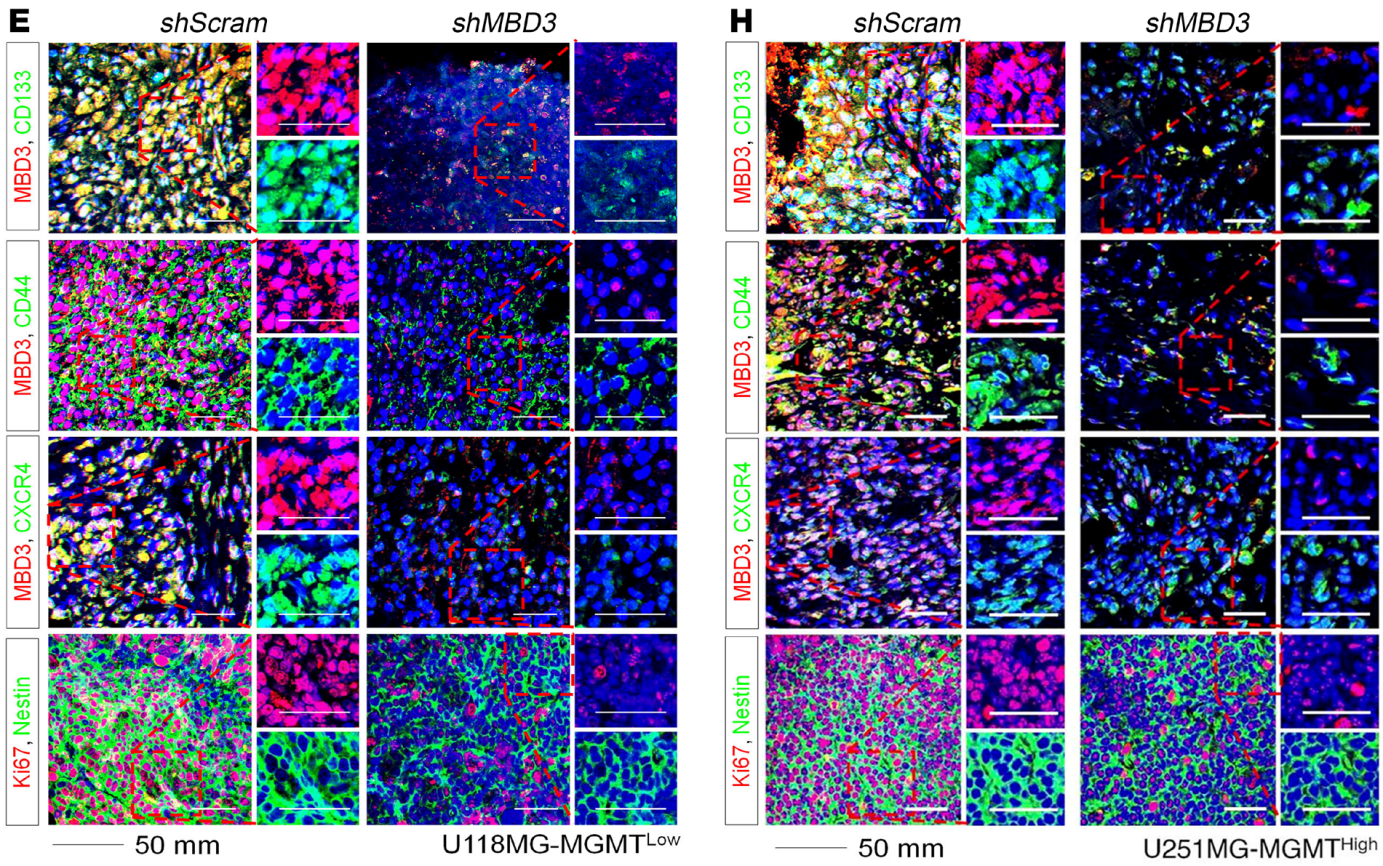# Corrigendum to Epigenetic modulator inhibition overcomes temozolomide chemoresistance and antagonizes tumor recurrence of glioblastoma

**DOI:** 10.1172/JCI196746

**Published:** 2025-08-01

**Authors:** Byoung-San Moon, Mingyang Cai, Grace Lee, Tong Zhao, Xiaofeng Song, Steven L. Giannotta, Frank J. Attenello, Min Yu, Wange Lu

Original citation: *J Clin Invest*. 2020;130(11):5782–5799. https://doi.org/10.1172/JCI127916

Citation for this corrigendum: *J Clin Invest*. 2025;135(15):e196746. https://doi.org/10.1172/JCI196746

In Figure 8E of the original article, there was an error in the shMBD3 CD133/MBD3 image, which was an inadvertent duplication of the image in Figure 9H for Pyr-Pam PDX CD133/MBD3. In addition, in Figure 8H, there was an error in the shScramble CD133/MBD3 image, which was an inadvertent duplication of the image in Figure 9H for TMZ PDX CD133/MBD3.

The corrected figure panels, based on the original source data, are provided below. The HTML and PDF versions of the paper have been updated.

Lastly, in Supplemental Figure 3E, the labels for CXCR4 and CD133 were swapped. The authors have updated the Supplemental Data file to correct the labels in Supplemental Figure 3E and to include disclosures in the figure legends explaining shared data in Supplemental Figures 2A, 2C, and 3E.

The authors regret the errors.

## Figures and Tables

**Figure 8, E and H:**